# The antioxidant responses of gills, intestines and livers and blood immunity of common carp (*Cyprinus carpio*) exposed to salinity and temperature stressors

**DOI:** 10.1007/s10695-022-01052-w

**Published:** 2022-02-16

**Authors:** Mahmoud A. O. Dawood, Mohamed Alkafafy, Hani Sewilam

**Affiliations:** 1 Animal Production Department, Faculty of Agriculture, Kafrelsheikh University, Kafr El-Sheikh, Egypt; 2 The Center for Applied Research On the Environment and Sustainability, The American University in Cairo, Cairo, 11835 Egypt; 3 Department of Biotechnology, College of Science, Taif University, P.O. Box 11099, Taif, 21944 Saudi Arabia; 4 Department of Engineering Hydrology, RWTH Aachen University, Aachen, Germany

**Keywords:** Hypersalinity, Thermal stress, Sustainable aquaculture, Oxidative stress, Immunity

## Abstract

Aquaculture activity is affected by various environmental factors, including water salinity and high temperatures. The present study investigated the impact of using varying water salinity (0, 5, 10, 15 and 20 ppt) on the growth behavior, immune responses and antioxidative responses of common carp. Fish were raised under optimal conditions except for water salinity for 8 weeks; fish were then subjected to high-temperature stress (32 °C) for 48 h. The results indicated a reduced final weight (FBW), weight gain (WG), specific growth rate (SGR), condition factor (CF), feed intake and feed efficiency ratio (FER) in common carp reared in 15 and 20 ppt (*p* < 0.05). The lowest FBW, WG, SGR, CF, feed intake and FER values were observed in fish reared in 20 ppt water salinity (*p* < 0.05). In gills, the superoxide dismutase (SOD), catalase (CAT) and glutathione peroxidase (GPx) were markedly decreased, but malondialdehyde (MDA) levels increased in fish challenged with 15 and 20 ppt before they were subjected to heat stress (*p* < 0.05). After heat stress, the SOD, CAT and GPx were decreased, and the MDA increased in fish reared in varying salinity levels (*p* < 0.05). Before heat stress, the intestinal SOD, CAT and GPx markers were decreased by 15 and 20 ppt, while the MDA level was increased by 15 and 20 ppt (*p* < 0.05). Generally, heat stress lowered the SOD, CAT and GPx activity in the intestines and liver tissues but increased MDA levels in common carp stressed by varying salinity levels (*p* < 0.05). The most decreased lysozyme activity, SOD, CAT and GPx and increased MDA levels were observed in common carp exposed to 20 ppt before and after heat stress (*p* < 0.05). After heat stress, fish exposed to 15 and 20 ppt had lower NBT than the remaining groups, and fish exposed to 20 ppt had the lowest values (*p* < 0.05). Overall, the heat stress markedly suppressed the antioxidant and immune responses of common carp reared in hypersalinity conditions.

## Introduction

Climatic changes are the primary reason for unstable environmental conditions in the aquaculture sector (Zarantoniello et al., 2021). The ecosystem is replete with components which have been directly impacted by climatic changes, such as water salinity and temperature (Imsland et al., 2003; Kim et al., 2017). Rivers and lakes are the major sources of water for fish farms, but increased temperatures have caused a high evaporation rate, especially during the summertime (Chang et al., 2021). Consequently, the level of water salinity has increased, leading to decreasing oxygen saturation and impacting osmoregulation (Dawood et al., 2020a, 2020c). Hypersalinity has harmful effects on growth behavior, osmoregulation, physiological status and immunological responses among freshwater species (Bu et al., 2021; Reza gholi tabar et al., 2020).

Common carp (*Cyprinus carpio*) is a widely cultured fish species with high market value (Abdel-Tawwab and Monier, 2018). Common carp grow mainly in freshwater conditions but can tolerate brackish conditions (Saravanan et al., 2018). However, hypersalinity impairs the fish’s osmoregulation capacity, producing several physiological problems and unstable health status (Evans and Kültz, 2020; Salati et al., 2011). Hypersalinity results in increased osmoregulation demands to adapt to stressful conditions that consume high energy levels, causing low feed consumption and growth rates (Saravanan et al., 2018; Tang et al., 2020). Several reports have investigated the impacts of hypersalinity on the performance of common carp (Naskar et al., 2021; Salati et al., 2011).

Hypersalinity and thermal stressors induce oxidative stress due to their effects on the induction of reactive oxygen species (ROS) involved in lipid peroxidation and cellular dysfunction (Sun et al., 2019a; Woo and Chung, 2020). Under oxidative stress, several markers exhibit deteriorated responses, including immune and antioxidative responses (Zhou et al., 2020). Several reports have elucidated the negative impact of hypersalinity and thermal stressors on the antioxidative and immune responses in finfish species (Abarike et al., 2020; Chen et al., 2021). Although many studies have investigated the impact of hypersalinity on the performance of common carp, minimal effort has been devoted to evaluating the combined effects of hypersalinity and temperature. Thus, this study explores the combined effects of hypersalinity and heat stress on the common carp’s growth behavior, antioxidative responses and immune responses. The results will help understand the physiological responses of common carp during stressful conditions to prevent the impact of climate change in fish farming.

## Materials and methods

### Experimental procedure

**Fig. 1:**
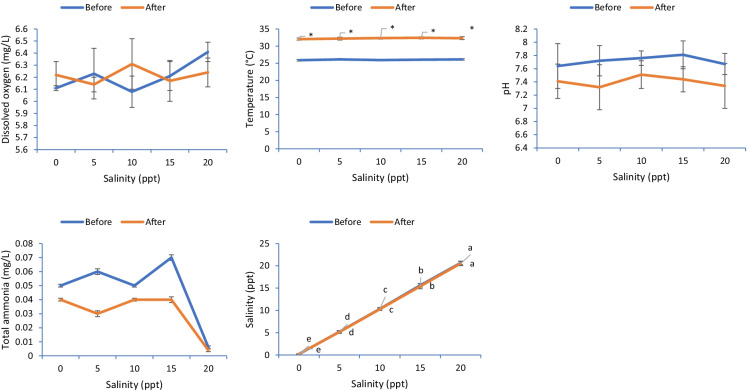
Water quality indices detected during the trial. Values ± standard error of mean (SEM) with different superscript letters refer to meaningful differences before or after the heat stress (*p* < 0.05). The symbol of “*” showing significant effect of heat stress on the measured indices (*p* < 0.05). Absence of different superscript letters refers to non-significant differences (*p* > 0.05)

Common carp juveniles were collected from a private farm located in Kafrelsheikh city, Egypt, and transported carefully to the Center for Applied Research on the Environment and Sustainability at The American University in Cairo, Egypt. Fish were placed in 1000- L plastic tanks and adapted to the lab conditions where the trial was undertaken for 2 weeks. The tank was provided with continuous aeration and freshwater that was partly exchanged daily. Fish were fed a commercial diet (30% crude protein, Skretting, Bilbis, El Sharqia Governorate, Egypt) twice daily at 3% of their body weight. Then, 225 fish of similar initial weight (6.57 ± 0.04 g/fish) were distributed in 15 plastic tanks (100 L) at 15 fish per tank (triplicates). Each tank was enriched with continuous aeration and freshwater (0 ppt). A stock tank with saline water was prepared daily by mixing sea salt with fresh water; the level of salinity was then gradually raised in the test tanks at 2 ppt on a daily basis except for the control. The salinity levels in the experimental tanks reached 0, 5, 10, 15 and 20 ppt through the daily process of water exchange. Fish were fed the commercial diet used during the adaptation period at 3% for 8 weeks twice daily (08:00 and 15:00). The water quality was checked using Martini Instruments Model 201/digital to detect the temperature, dissolved oxygen, salinity and pH (Fig. 1). The total ammonia level was detected calorimetrically using a HI-83303–02 Aquaculture Multi-parameter Photometer (Woonsocket, RI, USA).

### Final sampling

After 8 weeks, all fish fasted for 24 h before the final sampling. All fish were subsequently weighed and counted to calculate the specific growth rate and condition factor using the following equations:

Specific growth rate (SGR, % day) = ((Ln (FBW) − Ln (IBW)) / *t* (56 days)) × 100; weight gain (%) = ((FBW − IBW)/IBW) × 100; feed efficiency ratio (FER) = (FBW − IBW, g) / consumed feed (g); survival (%) = (final number of fish/initial number) × 100; condition factor (CF, %) = (FBW/L^3^) × 100.

IBW and FBW represent the initial and final body weight (g) of fish, respectively, and represent the number of days.

### Blood sampling and dissection

Three fish per tank were gently bled from the caudal vein in 2.5-mL tubes using non-heparinised syringes,”” and blood was collected for serum separation. Samples were left for 4 h at 4 °C and then centrifuged at 3000 × g for 15 min under 4 °C for serum collection. Serum samples were kept at − 80 °C for further biochemical analysis. Another two fish per tank were collected to dissect gills, intestines and livers to prepare the homogenate. The collected tissues were subsided in Eppendorf (2 mL) and then stored at − 20 °C for further processing. The homogenates were prepared by rinsing the gills in ice-cold phosphate-buffered saline (PBS) (pH 7.5; 1 g per 10 mL). It was then homogenized and centrifuged at 8000 rpm for 5 min, and the supernatant was collected and stored at 4 °C for further analysis.

### Antioxidative capacity

Superoxide dismutase, catalase and glutathione peroxidase activities in gills, intestines and liver homogenate samples were measured using diagnostic reagent kits following the manufacturer’s (Cusabio Biotech Co., Ltd., China) instructions. The concentration of malondialdehyde (MDA) was detected by adhering to the advice of Uchiyama and Mihara (1978) and expressed as nmol MDA/g. Briefly, gills, intestines and liver homogenates (10%, w/v) were mixed with 1.5 mL of 1% H_3_PO_4_ and 0.5 mL of 0.6% thiobarbituric acid. The tubes were heated for 60 min in a boiling water bath. After cooling in an ice bath, 2 mL of butanol was added, and contents were mixed vigorously for 20 s. After centrifugation (1107 g, 15 min), the absorbance of the organic layer was measured at 520 and 535 nm using a spectrometer (Lambda 2S, Perkin-Elmer Co., USA).

### Immune response

Serum lysozyme activity was determined using turbidimetric assay, according to the method described by Ellis (1990) based on the lysis of *Micrococcus lysodeikticus* (Sigma, USA). Briefly, a standard suspension of 0.15 mg/mL of *M. lysodeikticus* was prepared in 66 mM phosphate buffer (pH 6.0). Serum (50 μL) was then added to 1 mL of the bacterial suspension, and the absorbance reduction was recorded at 30-s and 4.5-min intervals at 450 nm using a spectrophotometer (SHIMADZU UV-1600PC). One unit of lysozyme was defined as reduction in absorbance of 0.001/min.

Blood respiratory burst activity was measured using a nitro-blue-tetrazolium (NBT) assay based on the advice of Secombes (1990). Briefly, 100 μL of the blood samples was added to each well of 96-well microtitre plate (Nalge-Nunc, Hereford, UK). The plate was incubated at 25 °C for 2 h to allow the attachment of the cells. Unattached cells were washed off three times using a fresh L-15 medium. The L-15 medium was then supplemented with NBT (1 mg/mL) and phorbol 12-myristate 13-acetate (PMA, Sigma-Aldrich; one μg/mL) dissolved in dimethyl sulphoxide (DMSO, Sigma), and 100 μL was added to each well of a microtitre plate and incubated for 1 h at room temperature. After incubation, the supernatant was removed from the plate, and NBT reduction was fixed with 100% methanol for 10 min. The plate was then washed with 70% methanol and left to air dry. A mixture of 120 μL of 2 M potassium hydroxide and 140 μL DMSO was added to dissolve the resulting formazan blue crystals. The NBT reduction was measured using the microplate reader (Optica, Mikura Ltd, UK) at 630 nm, and respiratory burst activity was expressed as NBT reduction.

### Heat stress

After the final sampling, the remaining fish (8 fish/tank) were kept in the same tanks equipped with electric heaters. The heat stress was performed by adhering to the practices of Dawood et al. (2020b). The water temperature level was raised gradually at 2 °C per hour until reaching 32 °C in all tanks, and the trial was run for 48 h under the same conditions followed during the first trial. The water quality was checked to confirm the experimental conditions using the same procedure as the first trial. After the heat stress, blood samples and dissection of gills, intestines and livers were executed using the same procedure mentioned earlier.

### Statistical analysis

Normality and homoscedasticity analyses were adopted before applying one-way variance (ANOVA) analysis for all groups using SPSS 22.0 software. Duncan’s multiple range test was then used to measure statistical differences among treatments, with the significance level set at *p* < 0.05. When significant differences were detected, a two-way ANOVA test was used to determine the effects of water salinity and heat stress and their interaction on common carp.

## Results

### Water quality

No marked effects were observed on the water temperature, dissolved oxygen, pH and total ammonia before the heat stress (*p*˃ 0.05). However, the water salinity level was increased gradually based on the proposed levels before the heat stress (*p* < 0.05). After heat stress, meaningful changes were observed in the water temperature compared to the water condition before heat stress (*p* < 0.05) (Fig. 1).

### Growth behavior

The final body weight (FBW), weight gain (WG) and specific growth rate (SGR) were all significantly reduced in fish reared in 15 and 20 ppt compared with fish grown in 0 and 5 ppt (*p* < 0.05). Fish reared in 10 ppt water salinity had no significant differences for FBW, WG and SGR relative to fish reared in 0 and 5 ppt (*p*˃ 0.05) (Table 1). The condition factor (CF) was lowered substantially in fish subjected to 10, 15 and 20 ppt water salinity compared with fish reared in 0 ppt salinity (*p* < 0.05) (Table 1). Additionally, fish reared in 5 ppt salinity exhibited no significant differences relative to fish grown in 0 ppt (*p*˃ 0.05). The feed intake and feed efficiency ratio (FER) showed reduced values in fish reared in 20 ppt compared with fish grown in 0, 5 and 10 ppt salinity (*p* < 0.05). Fish reared in 15 ppt water salinity displayed no significant differences for feed intake and FER compared to fish reared in 0, 5 and 10 ppt (*p*˃ 0.05) (Table 1). The survival rate was reduced in fish reared in 15 and 20 ppt compared with fish grown in 0 and 5 ppt (*p* < 0.05). Furthermore, fish reared in 20 ppt water salinity had lower survival rates than fish reared in 15 ppt (*p*˃ 0.05) (Table 1). The lowest FBW, WG, SGR, CF, FER and survival values were observed in fish reared in 20 ppt water salinity (*p* < 0.05).

**Table 1:** Growth behavior of common carp reared in varied levels of salinities

	0 ppt	5 ppt	10 ppt	15 ppt	20 ppt
IBW (g)	6.45 ± 0.07	6.52 ± 0.07	6.50 ± 0.04	6.67 ± 0.00	6.43 ± 0.02
FBW (g)	27.29 ± 0.41a	27.34 ± 0.41a	26.93 ± 0.19ab	25.47 ± 0.85b	23.72 ± 0.75c
WG (%)	323.14 ± 4.68a	319.51 ± 4.81a	314.35 ± 0.51ab	282.05 ± 12.69b	268.58 ± 10.63c
SGR (%/day)	2.40 ± 0.02a	2.39 ± 0.02a	2.37 ± 0.07ab	2.23 ± 0.06b	2.17 ± 0.05c
FI (g/fish)	49.05 ± 1.80a	47.82 ± 0.88a	48.15 ± 0.46a	47.44 ± 2.33ab	43.85 ± 1.23b
FER	0.42 ± 0.02a	0.43 ± 0.03a	0.42 ± 0.04a	0.39 ± 0.01ab	0.36 ± 0.02b
Survival (%)	97.78 ± 2.22a	97.78 ± 2.22a	100.00 ± 0.00a	93.33 ± 3.85b	82.22 ± 2.22c
CF%	2.59 ± 0.08a	2.22 ± 0.03ab	1.98 ± 0.19b	1.98 ± 0.04c	1.92 ± 0.02d

Values with different letters are significant different (*p* < 0.05). IBW and FBW were initial and final body weight (g) of fish, respectively

*WG* weight gain, *SGR* specific growth rate, *FI* feed intake, *FER* feed efficiency ratio, *CF* condition factor

### Antioxidative stress in gills

**Fig. 2:**
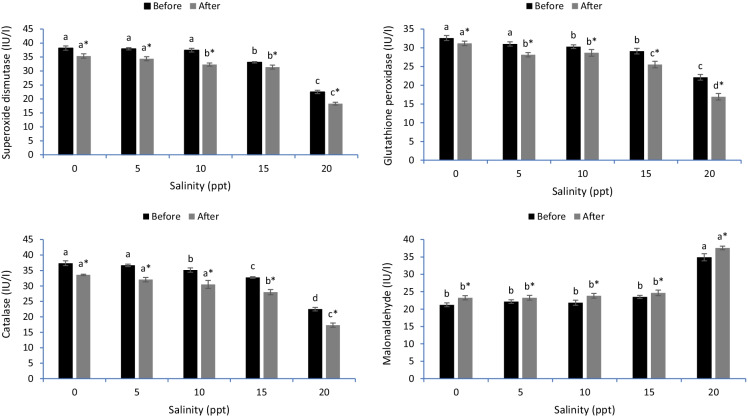
Gill antioxidant capacity of common carp reared in varied levels of salinities before and after heat stress. Bars ± standard error of mean (SEM) with different letters are significant different (*p* < 0.05) (*n* = 3). The symbol of “*” showing significant effect of heat stress on the measured indices (*p* < 0.05)

In gills, the superoxide dismutase (SOD), catalase (CAT) and glutathione peroxidase (GPx) were markedly decreased in fish subjected to 15 and 20 ppt salinity before the heat stress (*p* < 0.05) (Fig. 2). On the other hand, malondialdehyde (MDA) levels were significantly increased in fish stressed with 5, 10, 15 and 20 ppt, but no significant differences were observed between 5, 10 and 15 ppt (*p* < 0.05). After heat stress, the SOD, CAT and GPx were lowered, and the MDA increased in fish reared under varying salinity levels (*p* < 0.05). Before the heat stress, the 10, 15 and 20 ppt salinity levels decreased the SOD, CAT and GPx activities (*p* < 0.05). Before and after heat stress, the lowest SOD, CAT and GPx and the highest MDA level were detected in common carp raised in 20 ppt (*p* < 0.05) (Fig. 2).

### Intestine antioxidative stress

**Fig. 3:**
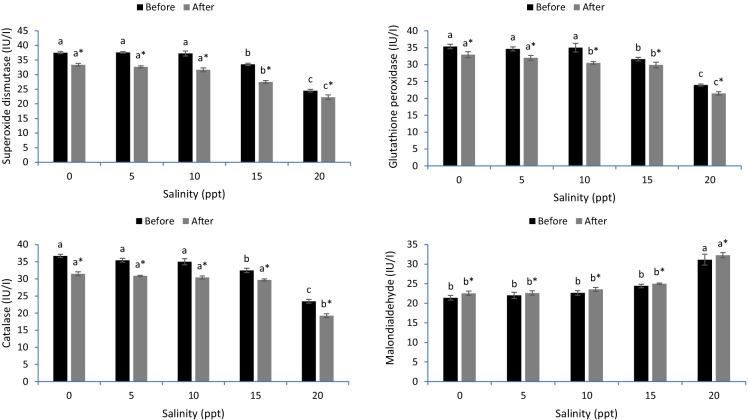
Intestinal antioxidant capacity of common carp reared in varied levels of salinities before and after heat stress. Bars ± standard error of mean (SEM) with different letters are significant different (*p* < 0.05) (*n* = 3). The symbol of “*” showing significant effect of heat stress on the measured indices (*p* < 0.05)

Before heat stress, the SOD, CAT and GPx markers were decreased by 15 and 20 ppt, while the MDA levels were increased by 15 and 20 ppt levels (*p* < 0.05) (Fig. 3). Generally, heat stress decreased the SOD, CAT and GPx activities but increased MDA levels in common carp stressed with varying salinity levels (*p* < 0.05). After heat stress, the activity of SOD was dramatically decreased in fish exposed to 15 and 20 ppt levels, and GPx was decreased under the 5, 10, 15 and 20 ppt levels (*p* < 0.05). The CAT activity was suppressed only in the case of 20 ppt salinity (*p* < 0.05). The level of MDA was markedly increased in the case of 15 and 20 ppt levels after heat stress (*p* < 0.05). In all cases, 15 to 20 ppt decreased the SOD, CAT and GPx activities and increased the MDA levels in common carp either before or after heat stress (*p* < 0.05).

### Liver antioxidative stress

**Fig. 4:**
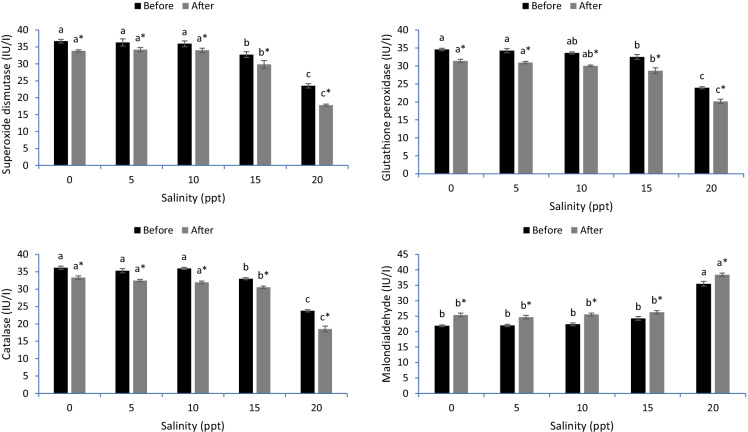
Liver antioxidant capacity of common carp reared in varied levels of salinities before and after heat stress. Bars ± standard error of mean (SEM) with different letters are significant different (*p* < 0.05) (*n* = 3). The symbol of “*” showing significant effect of heat stress on the measured indices (*p* < 0.05)

The antioxidative capacity of the liver tissue is illustrated in Fig. 4. Liver SOD exhibited lower activity in common carp stressed with 15 and 20 ppt relative to the other levels either before or after the heat stress (*p* < 0.05). The activity of GPx was decreased in fish exposed to 10, 15 and 20 ppt before and after heat stress (*p* < 0.05). CAT activity was decreased in fish exposed to 15 and 20 ppt before heat stress, but after heat stress, CAT activity was reduced in fish stressed with 5, 10, 15 and 20 ppt levels (*p* < 0.05). The MDA concentration was increased in fish exposed to 15 and 20 ppt before heat stress and 20 ppt after heat stress (*p* < 0.05). The most decreased SOD, CAT and GPx and increased MDA levels in the liver tissue were observed in common carp exposed to 20 ppt before and after heat stress (*p* < 0.05).

### Blood immunity

**Fig. 5:**
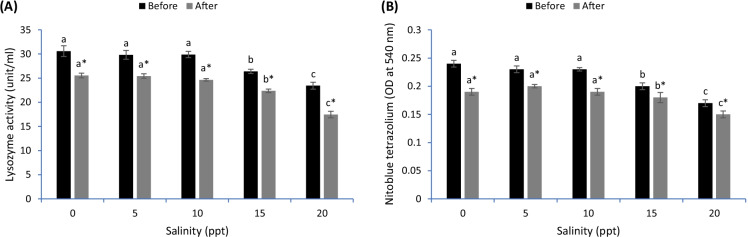
Blood immunity of common carp reared in varied levels of salinities before and after heat stress. Bars ± standard error of mean (SEM) with different letters are significant different (*p* < 0.05) (*n* = 3). The symbol of “*” showing significant effect of heat stress on the measured indices (*p* < 0.05)

Lysozyme activity was decreased in common carp exposed to 15 and 20 ppt compared with fish in the remaining groups before and after the heat stress (*p* < 0.05) (Fig. 5A). Additionally, fish of 20 ppt had lower lysozyme activity than fish of 15 ppt (*p* < 0.05). Before the heat stress, NBT was gradually decreased in fish subjected to varying salinity levels, and fish of 15 and 20 ppt had the lowest values (*p* < 0.05) (Fig. 5B). After heat stress, fish exposed to 15 and 20 ppt had lower NBT than the remaining groups, and fish of 20 ppt had the lowest values (*p* < 0.05) (Fig. 5B). Overall, the heat stress markedly decreased the lysozyme and NBT levels compared with fish before the heat stress (*p* < 0.05).

## Discussion

The optimal water salinity is required for proper growth behavior and production among finfish species (McCarthy et al., 2020). The results revealed that fish grew well up to 10 ppt, but the growth was markedly diminished in groups stressed with 15 and 20 ppt levels, as indicated by the measured FBW, WG and SGR. In this regard, Iffat et al. (2021) and Naskar et al. (2021) reported that common carp reared in brackish water (5 ppt) had normal growth performance, but hypersalinity (20 ppt) lowered the fish growth rate (Daudpota, 2020). It has been well established that fish are euryhaline and require optimal water salinity for normal growth and production (Chang et al., 2021; Mozanzadeh et al., 2021). This refers to the necessity of optimizing all farming conditions (e.g., temperature, ammonia and dissolved oxygen) to prevent the negative impact of brackish conditions on common carp growth performance. The reduced growth behavior of common carp under hypersalinity has been attributed to the impaired physiological and antioxidative stress resulting from hypersalinity (Luz et al., 2008). Indeed, hypersalinity reduces the feed intake and feed utilization, resulting in reduced digestibility and metabolic function (Rahmah et al., 2020). The lowered FBW, WG and SGR are also correlated with reduced feed intake and feed efficiency ratio (FER), which is significantly decreased in fish grown under hypersalinity (15 and 20 ppt). The reduction of FER results from low feed utilization in the intestines of common carp. As the primary organ responsible for osmoregulation, the intestine can lose its function in digesting and absorbing nutrients in the case of hypersalinity-induced oxidative stress (Dawood, 2021; Tian et al., 2020).

The results indicated a decreased survival rate in the groups of fish grown in 15 and 20 ppt. Unstable environmental conditions including hypersalinity impair the physiological status of fish (Sun et al., 2019b), leading to abnormal metabolic function and oxidative stress (Foyle et al., 2020). Similarly, low survival rates were observed in common carp stressed with salinity at 10 ppt (Abdel-Tawwab and Monier, 2018), 13 ppt (Roohi et al., 2017) and 15 ppt (Hoseinifar et al., 2014). The high mortality rate results from impaired physiological status, which correlates with the osmoregulation capacity of gills (Iffat et al., 2021). Consequently, fish grow slowly and suffer from high levels of mortality and diminished productivity.

Biometric somatic indices are visual indices correlated with the relative body weight and length (condition factor, CF) of fish (Mazumder et al., 2016). The measured CF index indicated reduced values in fish exposed to hypersalinity (10 and 15 ppt). The reduced CF refers to declining fish weight relative to the body length, indicating low weight gain and growth performance. Similarly, Kim et al. (2017) reported that sablefish (*Anoplopoma fimbria*) stressed by hypersalinity had a reduced CF index. The reduced CF is also related to the decreased fish growth under hypersalinity in the current trial conditions.

The cells of fish secrete antioxidative defenses such as superoxide dismutase (SOD), catalase (CAT) and glutathione peroxidase (GPx) to alleviate the impact of ROS on cellular function (Dawood et al., 2021). However, the long-term stress and severe conditions result in a high accumulation of ROS involved in lipid peroxidation, as evidenced by the high malondialdehyde (MDA) levels (Dragun et al., 2017). The results demonstrated lowered antioxidative indices (SOD, CAT and GPx) in the gills, livers and intestines of common carp stressed by hypersalinity (15 and 20 ppt) before heat stress. Furthermore, the thermal stress decreased SOD, CAT and GPx activity and increased MDA levels in the gills, liver and intestine tissues of common carp. The results are similar to those of Kim et al. (2017), who reported decreased SOD, CAT and GPx and increased MDA levels in sablefish exposed to hypersalinity. In another investigation, Wang et al. (2019) proved that thermal stress is responsible for oxidative stress in pikeperch (*Sander lucioperca*). However, no studies have uncovered the impact of both stressors on the antioxidative capacity of common carp. Kim et al. (2017) tested the combined effect of hypersalinity and thermal stressors on the antioxidative capacity of sablefish and revealed impaired antioxidative capacity with a high level of lipid peroxidation. The gills, intestine and liver tissues are the main organs involved in osmoregulation and resistance against oxidative stress induced by hypersalinity and high temperature (Foyle et al., 2020; Li et al., 2019). Hypersalinity disrupts the function of these organs, leading to disturbed osmoregulation and ion transfer (Salati et al., 2011). Additionally, hypersalinity and high temperature cause unstable respiration rates through the gills and low antioxidative capacity in the liver (Islam et al., 2020). Hypersalinity and thermal stressors impede intestinal digestion and local immunity, thereby suppressing the entire body’s immunity (Dawood, 2021). It is clear that hypersalinity and thermal stressors induce the increased generation of ROS (Madeira et al., 2013; Sinha et al., 2015), resulting in oxidative stress (Heise et al., 2006).

The results also indicate suppressed lysozyme activity in common carp exposed to hypersalinity prior to heat stress. Furthermore, there is a thermal effect on the activity of the lysozyme. These results are consistent with Kim et al. (2017), who discovered low lysozyme activity in sablefish exposed to hypersalinity and thermal stressors. There is a strong relationship between oxidative stress and suppressed immunity in aquatic animals (Paray et al., 2021). The accumulation of ROS induced by hypersalinity and thermal stressors causes dysfunction in the immune cells, leading to immunosuppression and a high possibility of infection from invaders (Shahjahan et al., 2020). Lysozyme activity is involved in the destruction of cell walls of harmful bacteria (Magnadóttir, 2006), but severe stressors, including hypersalinity and heat stress, act to lower lysozyme activity (Simide et al., 2016).

The present study showed decreased nitro-blue tetrazolium (NBT) levels in common carp exposed to hypersalinity, and high-temperature stressors indicated suppressed immunity. NBT is involved in the reaction with ROS produced by neutrophils to kill pathogenic bacteria as a non-specific immune response (Anderson, 1992). Thus, increased NBT is correlated with increased immunity and reduced NBT is associated with low immunity. Hypersalinity and thermal stressors are responsible for ROS generation in the entire bodies of fish, resulting in suppressed immunity and antioxidative capacity. Under hypersalinity, the gills, intestine and kidney organs suffer from stress, resulting in imbalanced ion gain and loss (Gui et al., 2016). More than 20–50% of the total energy budget of fish is consumed during osmoregulation (Arjona et al., 2009; Bœuf and Payan, 2001). Furthermore, the metabolic function and related physiological status exhibit unstable rates, leading to oxidative stress and immunosuppression (Freitas et al., 2017). The reduction of NBT is used to detect phagocytic respiratory burst activity, which is involved in the generation of ROS with high antibacterial activity (Buggé et al., 2007). NBT is also associated with metabolic activity in the electron transport system of the mitochondria. Although ROS results from other sources, the electron transport chain is still a significant source of ROS (Raha et al., 2000) because it generates a continuous flux of oxygen radicals (Petrosillo et al., 2003). Mitochondria consume 90% of the oxygen used by the cell, and 1–2% of all electrons crossing through the respiratory chain end up becoming oxygen radicals (Raha et al., 2000), which induce cellular oxidation and immune suppression.

## Conclusion

In conclusion, long-term hypersalinity reduced the growth behavior of common carp. Additionally, hypersalinity stress resulted in oxidative stress in the gills, intestines and livers of common carp, leading to decreased lysozyme activity and NBT levels. Exposure to thermal stress increased oxidative stress and diminished the immunity of common carp reared in hypersalinity conditions.

## Data Availability

The datasets are available from the corresponding author on reasonable request.

## References

[CR1] Abarike ED, Jian J, Tang J, Cai J, Sakyi EM, Kuebutornye FKA (2020). A mixture of Chinese herbs and a commercial probiotic Bacillus species improves hemato-immunological, stress, and antioxidant parameters, and expression of HSP70 and HIF-1α mRNA to hypoxia, cold, and heat stress in Nile tilapia Oreochromis niloticus. Aquaculture Reports.

[CR2] Abdel-Tawwab M, Monier MN (2018). Stimulatory effect of dietary taurine on growth performance, digestive enzymes activity, antioxidant capacity, and tolerance of common carp, *Cyprinus carpio* L., fry to salinity stress. Fish Physiol Biochem.

[CR3] Anderson DP (1992). Immunostimulants, adjuvants, and vaccine carriers in fish: applications to aquaculture. Annu Rev Fish Dis.

[CR4] Arjona FJ, Vargas-Chacoff L, Ruiz-Jarabo I, Gonçalves O, Páscoa I, Martín del Río MP, Mancera JM (2009). Tertiary stress responses in Senegalese sole (*Solea senegalensis* Kaup, 1858) to osmotic challenge: implications for osmoregulation, energy metabolism and growth. Aquaculture.

[CR5] Bœuf G, Payan P (2001). How should salinity influence fish growth?. Comp Biochem Physiol c: Toxicol Pharmacol.

[CR6] Bu X, Zhu J, Liu S, Wang C, Xiao S, Lu M, Li E, Wang X, Qin JG, Chen L (2021). Growth, osmotic response and transcriptome response of the euryhaline teleost, *Oreochromis mossambicus* fed different myo-inositol levels under long-term salinity stress. Aquaculture.

[CR7] Buggé DM, Hégaret H, Wikfors GH, Allam B (2007). Oxidative burst in hard clam (*Mercenaria mercenaria*) haemocytes. Fish Shellfish Immunol.

[CR8] Chang C-H, Wang Y-C, Lee T-H (2021). Hypothermal stress-induced salinity-dependent oxidative stress and apoptosis in the livers of euryhaline milkfish. Chanos chanos. Aquaculture.

[CR9] Chen Y, Liu E, Li C, Pan C, Zhao X, Wang Y, Ling Q (2021). Effects of heat stress on histopathology, antioxidant enzymes, and transcriptomic profiles in gills of pikeperch *Sander lucioperca*. Aquaculture.

[CR10] Daudpota AM (2020). Influence of salinity on growth increment, feed conversion and body composition of common carp, *Cyprinus carpio* (Linnaeus 1758) fingerlings in the captivity. Iran J Fish Sci.

[CR11] Dawood MAO (2021). Nutritional immunity of fish intestines: important insights for sustainable aquaculture. Rev Aquac.

[CR12] Dawood, M.A.O., Ali, M.F., Amer, A.A., Gewaily, M.S., Mahmoud, M.M., Alkafafy, M., Assar, D.H., Soliman, A.A., Van Doan, H., 2021. The influence of coconut oil on the growth, immune, and antioxidative responses and the intestinal digestive enzymes and histomorphometry features of Nile tilapia (*Oreochromis niloticus*). Fish Physiology and Biochemistry. 10.1007/s10695-021-00943-8 33770301

[CR13] Dawood MAO, Eweedah NM, El-Sharawy ME, Awad SS, Van Doan H, Paray BA (2020). Dietary white button mushroom improved the growth, immunity, antioxidative status and resistance against heat stress in Nile tilapia (*Oreochromis niloticus*). Aquaculture.

[CR14] Dawood MAO, Eweedah NM, Elbialy ZI, Abdelhamid AI (2020). Dietary sodium butyrate ameliorated the blood stress biomarkers, heat shock proteins, and immune response of Nile tilapia (*Oreochromis niloticus*) exposed to heat stress. J. Therm. Biol.

[CR15] Dawood MAO, Metwally AE-S, El-Sharawy ME, Ghozlan AM, Abdel-Latif HMR, Van Doan H, Ali MAM (2020). The influences of ferulic acid on the growth performance, haemato-immunological responses, and immune-related genes of Nile tilapia (*Oreochromis niloticus*) exposed to heat stress. Aquaculture.

[CR16] Dragun Z, Filipović Marijić V, Krasnići N, Ramani S, Valić D, Rebok K, Kostov V, Jordanova M, Erk M (2017). Malondialdehyde concentrations in the intestine and gills of Vardar chub (*Squalius vardarensis* Karaman) as indicator of lipid peroxidation. Environ Sci Pollut Res.

[CR17] Ellis AE (1990). Lysozyme Assays Techniques in Fish Immunology.

[CR18] Evans TG, Kültz D (2020). The cellular stress response in fish exposed to salinity fluctuations. Journal of Experimental Zoology Part a: Ecological and Integrative Physiology.

[CR19] Foyle, K.L., Hess, S., Powell, M.D., Herbert, N.A., 2020. What is gill health and what is its role in marine finfish aquaculture in the face of a changing climate? Frontiers in Marine Science 7.

[CR20] Freitas R, De Marchi L, Bastos M, Moreira A, Velez C, Chiesa S, Wrona FJ, Figueira E, Soares AMVM (2017). Effects of seawater acidification and salinity alterations on metabolic, osmoregulation and oxidative stress markers in *Mytilus galloprovincialis*. Ecol Ind.

[CR21] Gui L, Zhang P, Liang X, Su M, Wu D, Zhang J (2016). Adaptive responses to osmotic stress in kidney-derived cell lines from *Scatophagus argus*, a euryhaline fish. Gene.

[CR22] Heise K, Puntarulo S, Nikinmaa M, Abele D, Pörtner HO (2006). Oxidative stress during stressful heat exposure and recovery in the North Sea eelpout *Zoarces viviparus* L. J Exp Biol.

[CR23] Hoseinifar SH, Soleimani N, Ringø E (2014). Effects of dietary fructo-oligosaccharide supplementation on the growth performance, haemato-immunological parameters, gut microbiota and stress resistance of common carp (*Cyprinus carpio*) fry. Br J Nutr.

[CR24] Iffat J, Tiwari VK, Pavan-Kumar A, Verma AK, Harikrishna V, Babitha Rani AM, Chadha NK, Anand G (2021). The effect of inland saline groundwater on growth, maturation, and osmoregulation of common carp. N Am J Aquac.

[CR25] Imsland AK, Gunnarsson S, Foss A, Stefansson SO (2003). Gill Na+, K+-ATPase activity, plasma chloride and osmolality in juvenile turbot (*Scophthalmus maximus*) reared at different temperatures and salinities. Aquaculture.

[CR26] Islam MJ, Kunzmann A, Thiele R, Slater MJ (2020). Effects of extreme ambient temperature in European seabass, *Dicentrarchus labrax* acclimated at different salinities: growth performance, metabolic and molecular stress responses. Sci. Total Environ.

[CR27] Kim J-H, Park H-J, Kim K-W, Hwang I-K, Kim D-H, Oh CW, Lee JS, Kang J-C (2017). Growth performance, oxidative stress, and non-specific immune responses in juvenile sablefish, Anoplopoma fimbria, by changes of water temperature and salinity. Fish Physiol Biochem.

[CR28] Li C, Wang Y, Wang G, Chen Y, Guo J, Pan C, Liu E, Ling Q (2019). Physicochemical changes in liver and Hsc70 expression in pikeperch *Sander lucioperca* under heat stress. Ecotoxicol Environ Saf.

[CR29] Luz RK, Martínez-Álvarez RM, De Pedro N, Delgado MJ (2008). Growth, food intake regulation and metabolic adaptations in goldfish (*Carassius auratus*) exposed to different salinities. Aquaculture.

[CR30] Madeira D, Narciso L, Cabral HN, Vinagre C, Diniz MS (2013). Influence of temperature in thermal and oxidative stress responses in estuarine fish. Comp Biochem Physiol a: Mol Integr Physiol.

[CR31] Magnadóttir B (2006). Innate immunity of fish (overview). Fish Shellfish Immunol.

[CR32] Mazumder SK, Das SK, Bakar Y, Ghaffar MA (2016). Effects of temperature and diet on length-weight relationship and condition factor of the juvenile Malabar blood snapper (*Lutjanus malabaricus* Bloch & Schneider, 1801). Journal of Zhejiang University-SCIENCE B.

[CR33] McCarthy ID, Jones NJE, Moore DM, Berlinsky DL (2020). Determining the optimum temperature and salinity for larval culture, and describing a culture protocol for the conservation aquaculture for European smelt *Osmerus eperlanus* (L.). J Appl Ichthyol.

[CR34] Mozanzadeh MT, Safari O, Oosooli R, Mehrjooyan S, Najafabadi MZ, Hoseini SJ, Saghavi H, Monem J (2021). The effect of salinity on growth performance, digestive and antioxidant enzymes, humoral immunity and stress indices in two euryhaline fish species: yellowfin seabream (*Acanthopagrus latus*) and Asian seabass (*Lates calcarifer*). Aquaculture.

[CR35] Naskar S, Pailan GH, Datta S, Banerjee Sawant P, Bharti VS (2021). Effect of different organic manures and salinity levels on greenhouse gas emission and growth of common carp in aquaculture systems. Aquac Res.

[CR36] Paray BA, El-Basuini MF, Alagawany M, Albeshr MF, Farah MA, Dawood MA (2021). *Yucca schidigera* usage for healthy aquatic animals: potential roles for sustainability. Animals.

[CR37] Petrosillo G, Francesca MR, Di Venosa N, Paradies G (2003). Decreased complex III activity in mitochondria isolated from rat heart subjected to ischemia and reperfusion: role of reactive oxygen species and cardiolipin. FASEB J.

[CR38] Raha S, McEachern GE, Myint AT, Robinson BH (2000). Superoxides from mitochondrial complex III: the role of manganese superoxide dismutase. Free Radical Biol Med.

[CR39] Rahmah S, Liew HJ, Napi N, Rahmat SA (2020). Metabolic cost of acute and chronic salinity response of hybrid red tilapia *Oreochromis* sp. larvae. Aquaculture Reports.

[CR40] Reza gholitabar Z, Jafari V, Mazandarani M (2020). Effects of dietary Thyme (*Thymus vulgaris*) extract on the growth indices and some hematological parameters in common carp (*Cyprinus carpio*) to salinity stress. Journal of Applied Ichthyological Research.

[CR41] Roohi Z, Imanpoor MR, Jafari V, Taghizadeh V (2017). The use of fenugreek seed meal in fish diets: growth performance, haematological and biochemical parameters, survival and stress resistance of common carp (*Cyprinus carpio* L.). Aquac Res.

[CR42] Salati AP, Baghbanzadeh A, Soltani M, Peyghan R, Riazi G (2011). Effect of different levels of salinity on gill and kidney function in common carp *Cyprinus carpio* (Pisces: Cyprinidae). Italian Journal of Zoology.

[CR43] Saravanan M, Ramesh M, Petkam R, Poopal RK (2018). Influence of environmental salinity and cortisol pretreatment on gill Na+/K+-ATPase activity and survival and growth rates in *Cyprinus carpio*. Aquaculture Reports.

[CR44] Secombes, C.J.J.T.i.f.i., 1990. Isolation of salmonid macrophages and analysis of their killing activity. 137–154.

[CR45] Shahjahan, M., Khatun, M.S., Mun, M.M., Islam, S.M.M., Uddin, M.H., Badruzzaman, M., Khan, S., 2020. Nuclear and cellular abnormalities of erythrocytes in response to thermal stress in Common carp *Cyprinus carpio*. Frontiers in Physiology 11. 10.3389/fphys.2020.00543 PMC7289994 32581838

[CR46] Simide R, Richard S, Prévot-D’Alvise N, Miard T, Gaillard S (2016). Assessment of the accuracy of physiological blood indicators for the evaluation of stress, health status and welfare in Siberian sturgeon (*Acipenser baerii*) subject to chronic heat stress and dietary supplementation. International Aquatic Research.

[CR47] Sinha AK, AbdElgawad H, Zinta G, Dasan AF, Rasoloniriana R, Asard H, Blust R, De Boeck G (2015). Nutritional status as the key modulator of antioxidant responses induced by high environmental ammonia and salinity stress in European sea bass (*Dicentrarchus labrax*). PloS one.

[CR48] Sun J-L, Zhao L-L, Cui C, Du Z-J, He Z, Wang Y, Li X-W, Yang S (2019). Influence of long-term temperature stress on respiration frequency, Na+/K+-ATPase activity, and lipid metabolism in common carp (*Cyprinus carpio*). J Therm Biol.

[CR49] Sun J, Liu Q, Zhao L, Cui C, Wu H, Liao L, Tang G, Yang S, Yang S (2019). Potential regulation by miRNAs on glucose metabolism in liver of common carp (*Cyprinus carpio*) at different temperatures. Comparative Biochemistry and Physiology Part D: Genomics and Proteomics.

[CR50] Tang S, Liu S, Zhang J, Zhou L, Wang X, Zhao Q, Weng W, Qin JG, Chen L, Li E (2020). Relief of hypersaline stress in Nile tilapia *Oreochromis niloticus* by dietary supplementation of a host-derived *Bacillus subtilis* strain. Aquaculture.

[CR51] Tian L, Tan P, Yang L, Zhu W, Xu D (2020). Effects of salinity on the growth, plasma ion concentrations, osmoregulation, non-specific immunity, and intestinal microbiota of the yellow drum (*Nibea albiflora*). Aquaculture.

[CR52] Uchiyama M, Mihara M (1978). Determination of malonaldehyde precursor in tissues by thiobarbituric acid test. Anal Biochem.

[CR53] Wang Y, Li C, Pan C, Liu E, Zhao X, Ling Q (2019). Alterations to transcriptomic profile, histopathology, and oxidative stress in liver of pikeperch (*Sander lucioperca*) under heat stress. Fish Shellfish Immunol.

[CR54] Woo SJ, Chung JK (2020). Effects of trichlorfon on oxidative stress, neurotoxicity, and cortisol levels in common carp, *Cyprinus carpio* L., at different temperatures. Comparative Biochemistry and Physiology Part C. Toxicology & Pharmacology.

[CR55] Zarantoniello, M., Bortoletti, M., Olivotto, I., Ratti, S., Poltronieri, C., Negrato, E., Caberlotto, S., Radaelli, G., Bertotto, D., 2021. Salinity, temperature and ammonia acute stress response in Seabream (*Sparus aurata*) juveniles: a multidisciplinary study. Animals 11. 10.3390/ani11010097 PMC7825456 33419050

[CR56] Zhou L, Zhang J, Yan M, Tang S, Wang X, Qin JG, Chen L, Li E (2020). Inulin alleviates hypersaline-stress induced oxidative stress and dysbiosis of gut microbiota in Nile tilapia (*Oreochromis niloticus*). Aquaculture.

